# (Acetyl­acetonato-κ^2^ *O*,*O*′)chlorido­trimethano­latoniobium(V)

**DOI:** 10.1107/S1600536810021719

**Published:** 2010-06-16

**Authors:** Leandra Herbst, Renier Koen, Andreas Roodt, Hendrik G. Visser

**Affiliations:** a Department of Chemistry, University of the Free State, PO Box 339, Bloemfontein 9300, South Africa

## Abstract

In the title compound, [Nb(CH_3_O)_3_(C_5_H_7_O_2_)Cl], the Nb^V^ atom is coordinated by two O atoms from the chelating acetyl­acetonate ligand, three O atoms from the methano­late groups and one chloride ligand. The octa­hedral environment around niobium is slightly distorted with Nb—O distances in the range 1.8603 (15)–2.1083 (15) Å and an Nb—Cl distance of 2.4693 (9) Å. The O—Nb—O angles vary between 80.74 (6) and 100.82 (7)°, while the *trans* Cl—Nb—O angle is 167.60 (5)°. There are no hydrogen bonds observed, only an inter­molecular C—H⋯O inter­action.

## Related literature

For synthetic background, see: Davies *et al.* (1999 ▶). For applications of acetyl­acetone in industry, see: Steyn *et al.* (1992 ▶, 1997 ▶); Otto *et al.* (1998 ▶); Roodt & Steyn (2000 ▶); Brink *et al.* (2010 ▶); Viljoen *et al.* (2008 ▶, 2009*a* ▶,*b* ▶, 2010 ▶); Steyn *et al.* (2008 ▶). For related niobium complexes, see: Sokolov *et al.* (1999 ▶, 2005 ▶); Anti­nolo *et al.* (2000 ▶); Dahan *et al.* (1976 ▶).
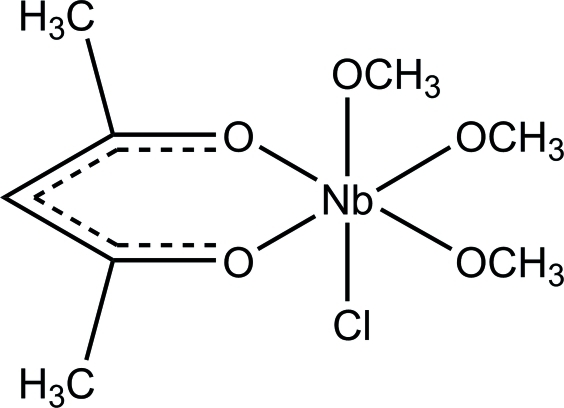


## Experimental

### 

#### Crystal data

[Nb(CH_3_O)_3_(C_5_H_7_O_2_)Cl]
*M* *_r_* = 320.57
Orthorhombic, 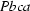

*a* = 12.296 (5) Å
*b* = 12.915 (4) Å
*c* = 15.470 (5) Å
*V* = 2456.7 (16) Å^3^
*Z* = 8
Mo *K*α radiation
μ = 1.20 mm^−1^
*T* = 100 K
0.36 × 0.30 × 0.19 mm

#### Data collection

Bruker X8 APEXII 4K Kappa CCD diffractometer
Absorption correction: multi-scan (*SADABS*; Bruker, 2004 ▶) *T*_min_ = 0.673, *T*_max_ = 0.805
28601 measured reflections
3083 independent reflections
2757 reflections with *I* > 2σ(*I*)
*R*_int_ = 0.030

#### Refinement

*R*[*F* ^2^ > 2σ(*F* ^2^)] = 0.023
*wR*(*F* ^2^) = 0.068
*S* = 1.16
3083 reflections
141 parameters
H-atom parameters constrained
Δρ_max_ = 1.06 e Å^−3^
Δρ_min_ = −0.87 e Å^−3^

### 

Data collection: *APEX2* (Bruker, 2005 ▶); cell refinement: *SAINT-Plus* (Bruker, 2004 ▶); data reduction: *SAINT-Plus*; program(s) used to solve structure: *SHELXS97* (Sheldrick, 2008 ▶); program(s) used to refine structure: *SHELXL97* (Sheldrick, 2008 ▶); molecular graphics: *DIAMOND* (Brandenburg & Putz, 2005 ▶); software used to prepare material for publication: *WinGX* (Farrugia, 1999 ▶).

## Supplementary Material

Crystal structure: contains datablocks global, I. DOI: 10.1107/S1600536810021719/pv2289sup1.cif

Structure factors: contains datablocks I. DOI: 10.1107/S1600536810021719/pv2289Isup2.hkl

Additional supplementary materials:  crystallographic information; 3D view; checkCIF report

## Figures and Tables

**Table:** 

O1—Nb1	1.8640 (15)
O2—Nb1	1.8811 (16)
O3—Nb1	1.8603 (15)
O4—Nb1	2.1083 (15)
O5—Nb1	2.0842 (15)
Cl1—Nb1	2.4693 (9)

**Table:** 

O3—Nb1—O1	100.82 (7)
O3—Nb1—O2	99.96 (7)
O1—Nb1—O2	99.45 (7)
O3—Nb1—O5	163.63 (6)
O1—Nb1—O5	91.53 (7)
O2—Nb1—O5	88.43 (7)
O3—Nb1—O4	85.71 (7)
O2—Nb1—Cl1	167.60 (5)

**Table 2:** Hydrogen-bond geometry (Å, °)

*D*—H⋯*A*	*D*—H	H⋯*A*	*D*⋯*A*	*D*—H⋯*A*
C8—H8*C*⋯O4^i^	0.98	2.46	3.442 (3)	176

Symmetry code: (i) 
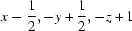
.

**Fig. 1.**
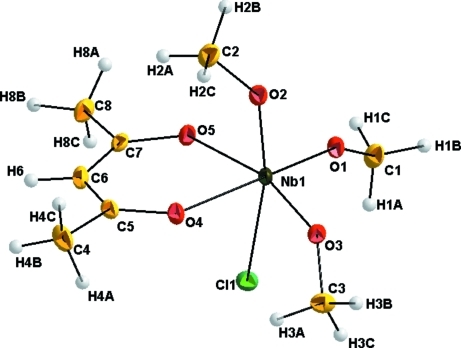
Molecular structure of the title compound. Displacement ellipsoids are drawn at the 50% probability displacement level.

## supplementary crystallographic information

### Comment

Acetylacetone and its analogues find applications in homogenous catalysis and the separations industry (Steyn *et al.*, 1992; 1997; Otto *et al.*, 1998; Roodt & Steyn, 2000; Brink *et al.*, 2010). This study forms part of ongoing research to investigate the intimate mechanism of the reactions of *O,O*'- and *N,O* -bidentate ligands with transition metals used in the nuclear industry, specifically hafnium, zirconium, niobium and tantalum (Viljoen *et al.*, 2008; 2009a,b; 2010; Steyn *et al.*, 2008).

Pale-yellow cubic crystals of the title complex crystallize from a methanol reaction solution containing niobium(V) chloride and acetylacetone after several days (Davies *et al.*, 1999). The asymmetric unit consists of a niobium(V) atom surrounded by three methanolate groups, a chloride ligand and a *O*,*O*'- bonded acetylacetonato ligand (Figure 1). The octahedral environment around niobium is slightly distorted with Nb–O distances varying between 1.8603 (15) and 2.1083 (15) Å, while the Nb–Cl distance is 2.4693 (9) Å. The O–Nb–O angles vary between 80.74 (6) and 100.82 (7) ° while the *trans* Cl–Nb–O angle is 167.60 (5) °. All the bond distances and angles are similar to other relevant niobium(V) structures (Sokolov *et al.*, 1999; 2005; Antinolo *et al.*, 2000 and Dahan *et al.*, 1976). The niobium compounds pack in a head-to-tail fashion along the *bc* plain.

There are no classical hydrogen bonds observed in this structure. However, the structure is stabilized by C8–H8C..O4* (* = -1/2+x,1/2-y,1-z) intermolecular interactions with C—H = 0.98, H···O = 2.46 and C···O = 3.442 (3) Å and C—H···O angle = 176°.

### Experimental

The reaction was performed under modified Schlenk conditions under an argon atmosphere. NbCl_5_ (0.3134 g, 1.16 mmol) was carefully dissolved in absolute methanol (5 ml) (Care: exothermic reaction). Acetylacetone (0.119 ml, 1.16 mmol) was added to the solution. The colourless solution was stirred for 1 h at room temperature and the solution was left to stand at 252 K for a few days after which pale-yellow crystals, suitable for X-ray diffraction were obtained.

### Refinement

The methyl and aromatic H atoms were placed in geometrically idealized positions and constrained to ride on their parent atoms, with C—H = 0.95 and 0.98Å and *U*_iso_(H) = 1.5*U*_eq_(C) and 1.2*U*_eq_(C), respectively. The highest residual electron-density peak is 0.93 Å from Cl1.

### Crystal data

**Table:**

[Nb(CH_3_O)_3_(C_5_H_7_O_2_)Cl]	*F*(000) = 1296
*M**_r_* = 320.57	*D*_x_ = 1.733 Mg m^−^^3^
Orthorhombic, *P**b**c**a*	Mo *K*α radiation, λ = 0.71073 Å
Hall symbol: -P 2ac 2ab	Cell parameters from 9878 reflections
*a* = 12.296 (5) Å	θ = 2.6–28.4°
*b* = 12.915 (4) Å	µ = 1.20 mm^−^^1^
*c* = 15.470 (5) Å	*T* = 100 K
*V* = 2456.7 (16) Å^3^	Cuboid, pale-yellow
*Z* = 8	0.36 × 0.3 × 0.19 mm

### Data collection

**Table:**

Bruker X8 APEXII 4K Kappa CCD diffractometer	3083 independent reflections
Radiation source: fine-focus sealed tube	2757 reflections with *I* > 2σ(*I*)
graphite	*R*_int_ = 0.030
ω and φ scans	θ_max_ = 28.4°, θ_min_ = 2.6°
Absorption correction: multi-scan (*SADABS*; Bruker, 2004)	*h* = −12→16
*T*_min_ = 0.673, *T*_max_ = 0.805	*k* = −14→17
28601 measured reflections	*l* = −18→20

### Refinement

**Table:**

Refinement on *F*^2^	Primary atom site location: structure-invariant direct methods
Least-squares matrix: full	Secondary atom site location: difference Fourier map
*R*[*F*^2^ > 2σ(*F*^2^)] = 0.023	Hydrogen site location: inferred from neighbouring sites
*wR*(*F*^2^) = 0.068	H-atom parameters constrained
*S* = 1.16	*w* = 1/[σ^2^(*F*_o_^2^) + (0.0273*P*)^2^ + 3.5334*P*] where *P* = (*F*_o_^2^ + 2*F*_c_^2^)/3
3083 reflections	(Δ/σ)_max_ = 0.001
141 parameters	Δρ_max_ = 1.06 e Å^−^^3^
0 restraints	Δρ_min_ = −0.87 e Å^−^^3^
0 constraints	

### Special details

**Table:**

Experimental. The intensity data were collected on a Bruker X8 ApexII 4 K Kappa CCD diffractometer using an exposure time of 60 s/frame. A total of 688 frames were collected with a frame width of 0.5° covering up to θ = 28.24° with 99.1% completeness accomplished.
Geometry. All s.u.'s (except the s.u. in the dihedral angle between two l.s. planes) are estimated using the full covariance matrix. The cell s.u.'s are taken into account individually in the estimation of s.u.'s in distances, angles and torsion angles; correlations between s.u.'s in cell parameters are only used when they are defined by crystal symmetry. An approximate (isotropic) treatment of cell s.u.'s is used for estimating s.u.'s involving l.s. planes.
Refinement. Refinement of *F*^2^ against ALL reflections. The weighted *R*-factor *wR* and goodness of fit *S* are based on *F*^2^, conventional *R*-factors *R* are based on *F*, with *F* set to zero for negative *F*^2^. The threshold expression of *F*^2^ > 2σ(*F*^2^) is used only for calculating *R*-factors(gt) *etc*. and is not relevant to the choice of reflections for refinement. *R*-factors based on *F*^2^ are statistically about twice as large as those based on *F*, and *R*- factors based on ALL data will be even larger.

### Fractional atomic coordinates and isotropic or equivalent isotropic displacement parameters (Å^2^)

**Table:**

	*x*	*y*	*z*	*U*_iso_*/*U*_eq_	
C1	0.00363 (19)	0.09540 (17)	0.76165 (14)	0.0195 (4)	
H1A	−0.0385	0.1594	0.7676	0.029*	
H1B	0.0228	0.0692	0.8191	0.029*	
H1C	−0.04	0.0435	0.7311	0.029*	
C2	0.3610 (2)	0.07674 (19)	0.54112 (15)	0.0217 (5)	
H2A	0.3222	0.0834	0.4861	0.033*	
H2B	0.3847	0.0049	0.5488	0.033*	
H2C	0.4247	0.1224	0.5409	0.033*	
C3	0.26952 (19)	0.38799 (16)	0.74055 (15)	0.0195 (4)	
H3A	0.2855	0.43	0.6894	0.029*	
H3B	0.3309	0.3915	0.7809	0.029*	
H3C	0.2039	0.4145	0.7689	0.029*	
C4	0.2957 (2)	0.37805 (19)	0.41139 (16)	0.0231 (5)	
H4A	0.3083	0.44	0.4466	0.035*	
H4B	0.2605	0.3978	0.357	0.035*	
H4C	0.3654	0.3443	0.399	0.035*	
C5	0.22378 (18)	0.30466 (16)	0.45973 (14)	0.0158 (4)	
C6	0.14339 (18)	0.24919 (17)	0.41581 (14)	0.0174 (4)	
H6	0.1304	0.2657	0.3569	0.021*	
C7	0.08152 (17)	0.17147 (16)	0.45334 (13)	0.0148 (4)	
O1	0.09963 (13)	0.11572 (11)	0.71426 (9)	0.0162 (3)	
O2	0.29097 (12)	0.10497 (12)	0.60982 (10)	0.0161 (3)	
O3	0.25251 (13)	0.28363 (11)	0.71528 (9)	0.0158 (3)	
O4	0.24286 (13)	0.29502 (11)	0.54083 (10)	0.0155 (3)	
O5	0.09160 (13)	0.14211 (11)	0.53257 (9)	0.0158 (3)	
Cl1	0.02912 (4)	0.32547 (4)	0.64105 (3)	0.01788 (11)	
Nb1	0.178955 (15)	0.198214 (14)	0.638103 (11)	0.01144 (7)	
C8	−0.00329 (19)	0.11656 (18)	0.40125 (14)	0.0200 (4)	
H8A	−0.0015	0.0424	0.4147	0.03*	
H8B	0.0113	0.1268	0.3396	0.03*	
H8C	−0.0752	0.1445	0.4154	0.03*	

### Atomic displacement parameters (Å^2^)

**Table:**

	*U*^11^	*U*^22^	*U*^33^	*U*^12^	*U*^13^	*U*^23^
C1	0.0207 (11)	0.0218 (10)	0.0160 (10)	−0.0056 (9)	0.0046 (8)	−0.0002 (8)
C2	0.0221 (11)	0.0254 (11)	0.0175 (10)	0.0029 (9)	0.0043 (9)	−0.0018 (8)
C3	0.0185 (11)	0.0155 (9)	0.0245 (11)	−0.0016 (8)	−0.0015 (9)	−0.0023 (8)
C4	0.0237 (12)	0.0247 (12)	0.0209 (11)	−0.0011 (9)	0.0053 (9)	0.0094 (9)
C5	0.0175 (10)	0.0160 (10)	0.0139 (10)	0.0040 (8)	0.0042 (8)	0.0037 (7)
C6	0.0192 (10)	0.0226 (10)	0.0105 (9)	0.0033 (9)	0.0009 (8)	0.0023 (8)
C7	0.0155 (10)	0.0178 (9)	0.0112 (9)	0.0054 (8)	−0.0003 (8)	−0.0029 (7)
O1	0.0187 (8)	0.0174 (7)	0.0125 (7)	−0.0016 (6)	0.0030 (6)	0.0022 (6)
O2	0.0161 (7)	0.0180 (7)	0.0140 (7)	0.0021 (6)	0.0020 (6)	0.0010 (6)
O3	0.0194 (8)	0.0148 (7)	0.0132 (7)	−0.0018 (6)	−0.0011 (6)	−0.0007 (5)
O4	0.0173 (7)	0.0170 (7)	0.0123 (7)	−0.0024 (6)	0.0009 (6)	0.0025 (5)
O5	0.0198 (8)	0.0165 (7)	0.0112 (7)	−0.0032 (6)	−0.0013 (6)	−0.0006 (5)
Cl1	0.0170 (2)	0.0169 (2)	0.0198 (3)	0.00236 (19)	0.00092 (19)	0.00026 (18)
Nb1	0.01376 (11)	0.01214 (10)	0.00841 (10)	−0.00081 (6)	0.00045 (6)	0.00115 (6)
C8	0.0199 (11)	0.0250 (11)	0.0150 (10)	0.0007 (9)	−0.0035 (8)	−0.0043 (8)

### Geometric parameters (Å, °)

**Table:**

C1—O1	1.414 (3)	C5—O4	1.282 (3)
C1—H1A	0.98	C5—C6	1.397 (3)
C1—H1B	0.98	C6—C7	1.387 (3)
C1—H1C	0.98	C6—H6	0.95
C2—O2	1.416 (3)	C7—O5	1.289 (3)
C2—H2A	0.98	C7—C8	1.497 (3)
C2—H2B	0.98	O1—Nb1	1.8640 (15)
C2—H2C	0.98	O2—Nb1	1.8811 (16)
C3—O3	1.419 (2)	O3—Nb1	1.8603 (15)
C3—H3A	0.98	O4—Nb1	2.1083 (15)
C3—H3B	0.98	O5—Nb1	2.0842 (15)
C3—H3C	0.98	Cl1—Nb1	2.4693 (9)
C4—C5	1.497 (3)	C8—H8A	0.98
C4—H4A	0.98	C8—H8B	0.98
C4—H4B	0.98	C8—H8C	0.98
C4—H4C	0.98		
			
O1—C1—H1A	109.5	O5—C7—C6	123.9 (2)
O1—C1—H1B	109.5	O5—C7—C8	116.1 (2)
H1A—C1—H1B	109.5	C6—C7—C8	120.0 (2)
O1—C1—H1C	109.5	C1—O1—Nb1	150.52 (14)
H1A—C1—H1C	109.5	C2—O2—Nb1	141.71 (14)
H1B—C1—H1C	109.5	C3—O3—Nb1	144.27 (14)
O2—C2—H2A	109.5	C5—O4—Nb1	133.45 (14)
O2—C2—H2B	109.5	C7—O5—Nb1	133.79 (14)
H2A—C2—H2B	109.5	O3—Nb1—O1	100.82 (7)
O2—C2—H2C	109.5	O3—Nb1—O2	99.96 (7)
H2A—C2—H2C	109.5	O1—Nb1—O2	99.45 (7)
H2B—C2—H2C	109.5	O3—Nb1—O5	163.63 (6)
O3—C3—H3A	109.5	O1—Nb1—O5	91.53 (7)
O3—C3—H3B	109.5	O2—Nb1—O5	88.43 (7)
H3A—C3—H3B	109.5	O3—Nb1—O4	85.71 (7)
O3—C3—H3C	109.5	O1—Nb1—O4	170.09 (6)
H3A—C3—H3C	109.5	O2—Nb1—O4	86.60 (7)
H3B—C3—H3C	109.5	O5—Nb1—O4	80.74 (6)
C5—C4—H4A	109.5	O3—Nb1—Cl1	87.49 (6)
C5—C4—H4B	109.5	O1—Nb1—Cl1	88.76 (5)
H4A—C4—H4B	109.5	O2—Nb1—Cl1	167.60 (5)
C5—C4—H4C	109.5	O5—Nb1—Cl1	82.03 (5)
H4A—C4—H4C	109.5	O4—Nb1—Cl1	84.06 (5)
H4B—C4—H4C	109.5	C7—C8—H8A	109.5
O4—C5—C6	123.7 (2)	C7—C8—H8B	109.5
O4—C5—C4	116.2 (2)	H8A—C8—H8B	109.5
C6—C5—C4	120.0 (2)	C7—C8—H8C	109.5
C7—C6—C5	123.8 (2)	H8A—C8—H8C	109.5
C7—C6—H6	118.1	H8B—C8—H8C	109.5
C5—C6—H6	118.1		
			
O4—C5—C6—C7	−5.6 (3)	C1—O1—Nb1—Cl1	5.1 (3)
C4—C5—C6—C7	172.5 (2)	C2—O2—Nb1—O3	−109.4 (2)
C5—C6—C7—O5	0.0 (3)	C2—O2—Nb1—O1	147.7 (2)
C5—C6—C7—C8	179.7 (2)	C2—O2—Nb1—O5	56.4 (2)
C6—C5—O4—Nb1	3.5 (3)	C2—O2—Nb1—O4	−24.4 (2)
C4—C5—O4—Nb1	−174.66 (15)	C2—O2—Nb1—Cl1	16.8 (4)
C6—C7—O5—Nb1	8.1 (3)	C7—O5—Nb1—O3	26.9 (3)
C8—C7—O5—Nb1	−171.59 (14)	C7—O5—Nb1—O1	166.10 (19)
C3—O3—Nb1—O1	−120.8 (2)	C7—O5—Nb1—O2	−94.49 (19)
C3—O3—Nb1—O2	137.5 (2)	C7—O5—Nb1—O4	−7.67 (19)
C3—O3—Nb1—O5	17.5 (4)	C7—O5—Nb1—Cl1	77.56 (19)
C3—O3—Nb1—O4	51.7 (2)	C5—O4—Nb1—O3	−169.00 (19)
C3—O3—Nb1—Cl1	−32.5 (2)	C5—O4—Nb1—O2	90.7 (2)
C1—O1—Nb1—O3	92.3 (3)	C5—O4—Nb1—O5	1.77 (19)
C1—O1—Nb1—O2	−165.5 (3)	C5—O4—Nb1—Cl1	−81.08 (19)
C1—O1—Nb1—O5	−76.9 (3)		

### Hydrogen-bond geometry (Å, °)

**Table:**

*D*—H···*A*	*D*—H	H···*A*	*D*···*A*	*D*—H···*A*
C8—H8C···O4^i^	0.98	2.46	3.442 (3)	176

Symmetry codes: (i) *x*−1/2, −*y*+1/2, −*z*+1.
